# Novel insights into the molecular pathogenesis of *CYP4V2*-associated Bietti's retinal dystrophy

**DOI:** 10.1002/mgg3.109

**Published:** 2014-09-15

**Authors:** Galuh D N Astuti, Vincent Sun, Miriam Bauwens, Ditta Zobor, Bart P Leroy, Amer Omar, Bernhard Jurklies, Irma Lopez, Huanan Ren, Volkan Yazar, Christian Hamel, Ulrich Kellner, Bernd Wissinger, Susanne Kohl, Elfride De Baere, Rob W J Collin, Robert K Koenekoop

**Affiliations:** 1 Department of Human Genetics, Radboud University Medical CentreNijmegen, The Netherlands; 2 Radboud Institute for Molecular Life Sciences, Radboud University Medical CentreNijmegen, The Netherlands; 3 Division of Human Genetics, Center for Biomedical Research, Faculty of Medicine, Diponegoro UniversitySemarang, Indonesia; 4 McGill Ocular Genetics Laboratory, Departments of Paediatric Surgery, Human Genetics and Ophthalmology, Montreal Children's Hospital, McGill University Health CentreMontreal, Quebec, Canada; 5 Center for Medical Genetics, Ghent University HospitalGhent, Belgium; 6 Institute for Ophthalmic Research, Centre for Ophthalmology, University of TübingenTübingen, Germany; 7 Department of Ophthalmology, Ghent University HospitalGhent, Belgium; 8 Moorfields Eye HospitalLondon, United Kingdom; 9 Department of Ophthalmology, University of EssenEssen, Germany; 10 Institute of Neurosciences of Montpellier, Hôpital Saint EloiMontpellier, France; 11 Rare Retinal Disease Center, AugenZentrum Siegburg, MVZ ADTC Siegburg GmbHSiegburg, Germany

**Keywords:** Bietti, crystalline dystrophy, *CYP4V2*, retinal dystrophy

## Abstract

Bietti's crystalline dystrophy (BCD) is a rare, autosomal recessive retinal degenerative disease associated with mutations in *CYP4V2*. In this study, we describe the genetic and clinical findings in 19 unrelated BCD patients recruited from five international retinal dystrophy clinics. Patients underwent ophthalmic examinations and were screened for *CYP4V2* mutations by Sanger sequencing and quantitative polymerase chain reaction (qPCR) copy number variation screening. Eight *CYP4V2* mutations were found in 10/19 patients, including three patients in whom only monoallelic mutations were detected. Four novel mutations were identified: c.604G>A; p.(Glu202Lys), c.242C>G; p.(Thr81Arg), c.604+4A>G; p.(?), and c.1249dup; p.(Thr417Asnfs*2). In addition, we identified a heterozygous paternally inherited genomic deletion of at least 3.8 Mb, encompassing the complete *CYP4V2* gene and several other genes, which is novel. Clinically, patients demonstrated phenotypic variability, predominantly showing choroidal sclerosis, attenuated vessels, and crystalline deposits of varying degrees of severity. To our knowledge, our study reports the first heterozygous *CYP4V2* deletion and hence a novel mutational mechanism underlying BCD. Our results emphasize the importance of copy number screening in BCD. Finally, the identification of *CYP4V2*-negative patients with indistinguishable phenotypes from *CYP4V2*-positive patients might suggest the presence of mutations outside the coding regions of *CYP4V2*, or locus heterogeneity, which is unreported so far.

## Introduction

Bietti crystalline dystrophy (BCD) (OMIM 210370) is a rare retinal degenerative disease that is inherited in an autosomal recessive pattern (Li et al. 2004). First described in three patients by the Italian ophthalmologist Bietti (1937) in Rome, BCD is defined and characterized by glistening crystalline deposits in the fundus associated with atrophy of the retinal pigment epithelium (RPE) and choroidal sclerosis (Bietti 1937; Rossi et al. 2013). Crystal deposits at the corneal limbus have also been documented (Rossi et al. 2013). Clinically, patients affected with BCD present between the second and fourth decade of life with impaired vision, nyctalopia, and paracentral scotomas. Vision loss and concentric visual field constriction continue progressively, often resulting in severe visual impairment by the fifth or sixth decade (Li et al. 2004; Lee et al. 2005).

BCD has been reported to be more prevalent in Asian populations, although patients of European, Middle Eastern, African, and North and South American origin have also been documented (Hu 1987; Li et al. 2004). Moreover, it has been estimated that up to 3% of patients initially diagnosed with nonsyndromic retinitis pigmentosa can be accounted for by BCD (Mataftsi et al. 2004). BCD is known to be caused by mutations in the *CYP4V2* gene, localized on chromosome 4q35 (Li et al. 2004). Currently, up to 57 mutations in *CYP4V2* have been associated with BCD; the majority are missense/nonsense mutations, as well as several small insertions, deletions, and splicing mutations (Li et al. 2004; Lee et al. 2005; Lin et al. 2005; Shan et al. 2005; Wada et al. 2005; Jin et al. 2006; Lai et al. 2007; Zenteno et al. 2008; Mamatha et al. 2011; Xiao et al. 2011; Yokoi et al. 2011; Haddad et al. 2012; Manzouri et al. 2012; Parravano et al. 2012; Song et al. 2013; Halford et al. 2014; Yin et al. 2014). *CYP4V2* encodes a member of the cytochrome P450 superfamily, characterized as a fatty acid oxidase involved in lipid metabolism (Nakano et al. 2009). Surprisingly, evidence of altered lipid metabolism has also been demonstrated in fibroblasts and lymphocytes of patients with BCD, in which synthesis of n-3 polyunsaturated fatty acids was decreased (Lee et al. 2001). Crystalline deposits have also been reported in these cells (Wilson et al. 1989). Furthermore, patients with BCD have been shown to have abnormal serum fatty acid profiles, with increased stearic acid and reduced oleic acid concentrations (Lai et al. 2010). These findings suggest that BCD represents a systemic condition that is not limited to the eye. However, it is still unclear whether these systemic abnormalities are actually disease-causing or whether they are subclinical (Li et al. 2004). It has been hypothesized that the retinal crystals represent lipids or fatty acids (Lai et al. 2010). Finally, the phenotypic spectrum of *CYP4V2* mutations has been expanded to autosomal recessive retinitis pigmentosa (arRP), without intraretinal crystals being observed in affected patients (Mataftsi et al. 2004).

In this study, we present novel genetic and clinical findings in a large international cohort of 19 probands from unrelated families with apparent Bietti crystalline retinal dystrophy.

## Materials and Methods

### Subject enrolment and clinical evaluation

Nineteen patients were recruited from and evaluated at the McGill Ocular Genetics Laboratory and Clinic at the McGill University Health Centre, in Montreal Canada; the Centre for Ophthalmology at the University of Tübingen, in Germany; the Ophthalmic Genetics Clinic at the Ghent University Hospital, in Belgium; the Rare Retinal Disease Centre (Siegburg Eye Center), Germany; and INSERM in Montpellier, France. Informed consent was obtained and research protocols adhered to the tenets of the Declaration of Helsinki. Institutional Review Board (IRB)/Ethics Committee approval was obtained. Patients had histories and pedigree analysis taken, and underwent ophthalmic examination including visual acuity (VA) testing, Goldmann visual field testing, refraction, slit lamp examination, dilated fundus examination, fundus photography, fundus autofluorescence (FAF), optical coherence tomography (OCT), and full-field flash electroretinography (ERG), recorded in accordance with the guidelines of the International Society for Clinical Electrophysiology of Vision (Marmor et al. 2009). One patient did not have Goldmann visual field testing nor OCT testing. In addition, fluorescein angiography was also performed in two patients. Phlebotomy in ethylenediaminetetraacetic acid tubes was performed to collect venous blood for genetic studies.

### Mutation analysis

#### Homozygosity mapping (only performed in Lebanese family A)

Genomic DNA was isolated from lymphocytes by standard salting out procedures (Miller et al. 1988). DNA samples of all four affected individuals from family A were genotyped on the GeneChip Genome-Wide Human SNP Array 5.0 that contains 500,000 polymorphic SNPs in addition to 420,000 nonpolymorphic probes for the detection of germline copy number variations (CNVs) (Affymetrix, Santa Clara, CA). Array experiments were performed according to protocols provided by the manufacturer. The 5.0 array data were genotyped using Affymetrix Genotype Console (version 2.1), subsequently regions of homozygosity were identified using Partek Genomics Solution (version 6.1), as described previously (Collin et al. 2011). Regions containing more than 250 consecutive homozygous SNPs were considered as homozygous regions, on average corresponding to a genomic size of 1 Mb or more.

#### Mutation analysis

All exons and intron–exon boundaries of *CYP4V2* (NM_207352) were amplified under standard polymerase chain reaction (PCR) conditions using primers listed in Table S1. PCR products were purified on Nucleospin Plasmid Quick Pure columns (Machery Nagel, Düren, Germany) and sequenced in sense and antisense directions with dye termination chemistry on a 3730 or 2100 DNA analyzer (Applied Biosystems, Carlsbad, CA).

#### Bioinformatic analysis and evolutionary comparison for missense mutations

For each of the missense changes identified in this study, the potential pathogenicity was assessed using online prediction software tools SIFT (Sorting Intolerant from Tolerant) and PolyPhen (Ng and Henikoff 2003). Grantham and PhyloP scores were also determined. In addition, the Exome Variant Server database was checked for the presence and minor allele frequencies of these novel mutations. Four computational programs, SpliceSite finder-like, MaxEntScan (Yeo and Burge 2004), NNSPLICE (Reese et al. 1997), and Human Splicing Finder (Desmet et al. 2009) were employed to predict the effect on the canonical acceptor and donor splice sites.

#### CNV screening using qPCR and SNP chip arrays

CNV screening on genomic DNA was performed in the families with only one *CYP4V2* mutation (probands F, I, and J), using 13 quantitative PCR (qPCR) assays, covering the 11 exons of *CYP4V2* and two reference genes (*ZNF80* and *GPR15*) on the LightCycler 480 (Roche, Basel, Switzerland). Four controls were included in each experiment. Conditions and primers can be found in Table S2. Data analysis was performed using qBasePlus (Biogazelle, Zwijnaarde, Belgium).

Genome-wide SNP chip analysis was performed using the HumanCytoSNP-12 BeadChip platform (Illumina, San Diego, CA) (proband F) in order to delineate the deletion.

## Results

### Patient demographics and genotyping

**Figure 1:**
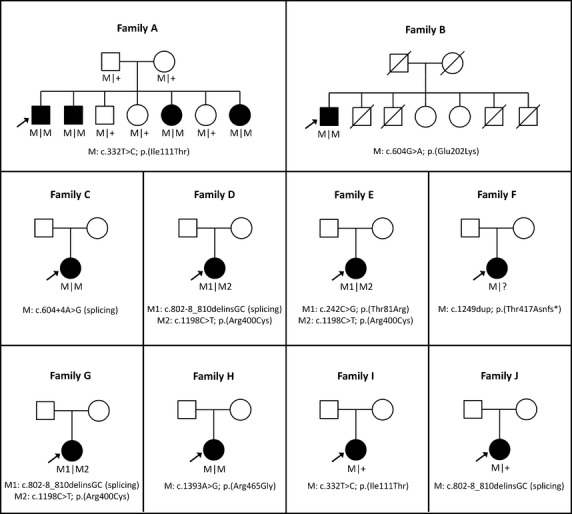
Pedigrees of 10 families with Bietti crystalline dystropy carrying mutations in *CYP4V2*. Affected individuals are indicated with filled symbols, whereas unaffected relatives are indicated by open symbols. The probands are indicated with an arrow and slashed symbols indicate deceased. Mutated alleles are indicated with M symbols and wild-type allele with plus symbols.

In a consanguineous Lebanese family that was initially diagnosed with atypical RP (Fig.1A), genome-wide SNP array analysis was combined with homozygosity mapping to identify genomic regions that could potentially harbor the causative genetic defect. In total, only two homozygous regions were identified that were identical between the four affected siblings, one of which harbored the *CYP4V2* gene, located on chromosome 4. Sequence analysis revealed a homozygous missense mutation in *CYP4V2*, c.332T>C; p. (Ile111Thr) that completely segregated in the family (Fig.1A). Most notably, the proband presented with severe choroidal sclerosis but no crystals were found on fundus examination. Upon further family examination, an affected younger sibling, however, was noted to have diffuse crystalline deposits in the fundus.

Subsequently, 18 additional patients were ascertained that were diagnosed with retinal dystrophy and presented or had presented with crystalline deposits. Sequence analysis of all exons and intron–exon boundaries of *CYP4V2* in this cohort revealed nine more patients with *CYP4V2* mutations, four carrying apparent homozygous mutations, three with compound heterozygous mutations, and two individuals with only one heterozygous intragenic variant (Fig.1). All these patients were of Caucasian origin.

Patients with *CYP4V2* mutations included seven females and three males, with ages ranging between 33 and 77 years. Initially, eight different mutations in *CYP4V2* were identified, including four previously documented and four novel mutations. The novel mutations were two missense, one splice site and one frameshift mutation; namely c.604G>A; p.(Glu202Lys), c.242C>G; p.(Thr81Arg), c.604+4A>G; p.(?), and c.1249dup; p.(Thr417Asnfs*2) (Table1). The novel missense mutations were not observed in the comprehensive Exome Variant Server database, while the already published variants p.(Arg400Cys) and p.(Arg465Gly) have been identified with low minor allele frequencies of 0.0154 and 0.0077, respectively. The most prevalent mutations identified in our cohort were the c.1198C>T; p.(Arg400Cys), c.332T>C; p.(Ile111Thr), and c.802-8_810delinsGC; p.(?) mutations, each accounting for 3 of the 18 mutant alleles identified in the probands. We found the p. Arg400Cys mutation in three patients, the p. Ile111Thr in two patients, and the c.802-8_810delinsGC; p.(?) change in three patients.

**Table 1:** Mutation table

Proband	Type	Allele 1		Allele 2	
A	Homozygous	c.332T>C	p.Ile111Thr	c.332T>C	p.Ile111Thr
B	Homozygous	c.604G>A	p.Glu202Lys	c.604G>A	p.Glu202Lys
C	Homozygous	c.604+4A>G	splicing	c.604+4A>G	Splicing
D	Compound heterozygous	c.802-8_810delinsGC	Splicing	c.1198C>T	p.Arg400Cys
E	Compound heterozygous	c.242C>G	p.Thr81Arg	c.1198C>T	p.Arg400Cys
F	Compound heterozygous	c.1249dup	p.Thr417Asnfs^*^2	Genomic deletion	Genomic deletion
G	Compound heterozygous	c.802-8_810delinsGC	Splicing	c.1198C>T	p.Arg400Cys
H	Homozygous	c.1393A>G	p.Arg465Gly	c.1393A>G	p.Arg465Gly
I	Heterozygous	c.332T>C	p.Ile111Thr	No second allele found		
J	Heterozygous	c.802-8_810delinsGC	splicing	No second allele found		

To evaluate the pathogenicity of the novel mutations, in silico analysis using a variety of prediction programs was performed. The two novel missense mutations, p.(Thr81Arg) and p.(Glu202Lys), affect amino acid residues that are highly conserved among vertebrate species. Both substitutions have a high Grantham score (Table2). Moreover, the p.(Glu202Lys) mutation, is predicted to be pathogenic by the two in silico programs SIFT and PolyPhen (Table2). The p.(Thr417Asnfs*2) frameshift mutation is presumed to create a premature stop codon one amino acid residue downstream, and in addition might be targeted for nonsense-mediated decay (NMD). In silico prediction of the c.604+4A>G splice site mutation showed a decrease in the strength of the splice donor site due to the alteration (e.g., 12% decrease in SpliceSite finder-like, 52% in MaxEntScan, 89.5% in NNSPLICE, and 9% decrease in Human Splicing Finder [HSF]), suggesting that this mutation might alter *CYP4V2* splicing.

**Table 2:** Pathogenicity predictions for missense and splice site mutations based on in silico analyses.

Amino acid change predictions							
DNA change	Mutation consequence	Exome variant server^1^	PhyloP	Grantham score	SIFT	PolyPhen	References
c.242C>G	p.(Thr81Arg)	–	2.55	71	Tolerated	Benign	Novel
c.332T>C	p.(Ile111Thr)	–	4.56	89	Deleterious	Probably damaging	Li et al. (2004)
c.604G>A	p.(Glu202Lys)	–	5.61	56	Deleterious	Probably damaging	Novel
c.1198C>T	p.(Arg400Cys)	2/13004 MAF 0.0154	4.48	180	Deleterious	Probably damaging	Lai et al. (2007)
c.1393A>G	p.(Arg465Gly)	2/13004 MAF 0.0077	1.25	125	Deleterious	Probably damaging	Rossi et al. (2013)

Splicing prediction							
DNA change	Mutation consequence	Position	SSF (0–100)	MaxEnt (0–12)	NNSPLICE (0–1)	HSF (0–100)	Reference
c.604+4A>G	Altered splicing	c.604	84.07 ⇒ 73.99 (−12.0%)	8.95 ⇒ 4.29 (−52.1%)	0.98 ⇒ 0 (−100%)	92.33 ⇒ 83.99 (−9.0%)	Novel

List of missense and splice site mutations identified in this study and predictions of their consequences with the use of in silico program (SIFT and PolyPhen). Splicing prediction shows the percent decrease in comparison to the original splice donor site scores. In addition, HSF predicted a novel splice donor site in position c.604+4. SIFT, sorting intolerant from tolerant; MAF, minor allele frequency; HSF, Human Splicing Finder; SSF, Splice Sequence Finder; NNSPLICE, Splice Site Prediction by Neural Network.

1 Heterozygous alleles of total number of chromosomes.

**Figure 2:**
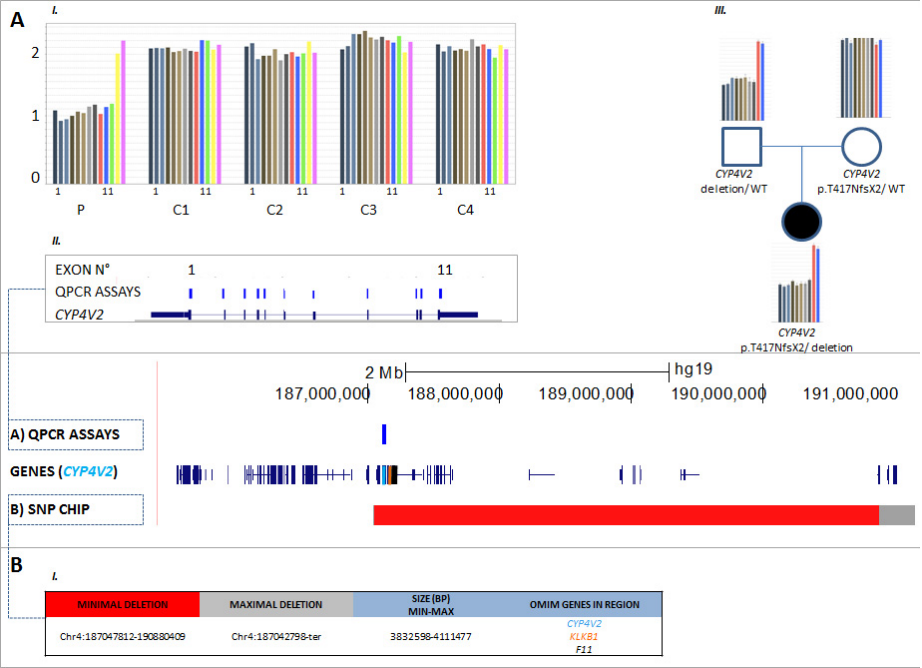
Detection and delineation of a *CYP4V2* deletion in proband F using qPCR and SNP-chip analysis. C (control), P (patient), WT (wild type). (A) I and II: qPCR on proband F revealed a copy number of one for all 11 assays of *CYP4V2*, corresponding with a heterozygous total gene deletion. The same was found for the other deleted genes; KLKB1 and F11. III: Demonstration of paternal origin of the deletion. (B) I: SNP-chip analysis demonstrates deletion with size ∼4 Mb, spanning the entire *CYP4V2* gene and several other genes, two of which are OMIM genes (*KLKB1* and *F11*).

Upon assessing the segregation of mutations in available family members, an inconsistency was noted for proband F, who carried an apparent homozygous mutation c.1249dup; p.(Thr417Asnfs*2). The mutation was found to segregate in the patient's mother however not in her father, while paternity was confirmed. Subsequently, genomic qPCR analysis revealed a heterozygous deletion encompassing all exons of *CYP4V2*, in both proband F and her father (Fig.2A). To further delineate the breakpoints and determine the extent of this new deletion, SNP-chip analysis was performed. The deletion size was demonstrated to vary between 3.8 and 4.1 Mb, spanning the entire *CYP4V2* gene and several other genes, including two OMIM genes (*KLKB1* and *F11*) (Fig.2B). No *CYP4V2* deletions were detected in the two other probands who carried a single heterozygous allele (data not shown).

### Patient phenotypes

**Figure 3:**
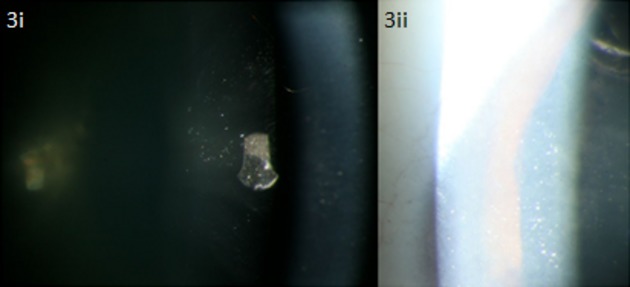
Crystalline deposits seen on the lens (i) and corneal limbus (ii) of 54-year-old proband D with compound heterozygous mutations in *CYP4V2* c.802-8_810delinsGC; p.(?), p.(Arg400Cys).

The clinical data for all 10 patients that carried one or two *CYP4V2* alleles are described in Table3. Disease onset in these patients ranged from 18 to 57 years of age, with duration of illness ranging from 8 to 33 years. Visual acuities (VA) varied extensively and ranged from 20/20 to light perception (LP), with large discrepancies in VA between both eyes occasionally noted. With the exception of three patients with no refractive error, all subjects were myopic with astigmatism with refraction ranging from −0.50 to −6.00 dioptres. Goldmann visual fields also revealed a wide spectrum of severity, ranging from relatively normal to severe constriction (5–10° remaining). Central, paracentral, and ring scotomas were also noted. Crystalline deposits were noted in the corneal limbus of three patients. In proband D, crystalline deposits were seen on the lens as well as the limbus (Fig.3). Posterior subcapsular cataracts (PSCC) and cortical opacities were noted in several patients.

**Table 3:** Clinical features of 10 BCD patients with mutations in *CYP4V2*.

					Visual acuity						Visual field			
Family	ID	Age	Age at onset	Gender	Initial visual acuity (OD)	Initial visual acuity (OS)	Visual Acuity (OD)	Visual Acuity (OS)	Refraction (OD)	Refraction (OS)	Initial visual field (OD)	Initial visual field (OS)	Visual field (OD)	Visual field (OS)
A	MOGL 3254	57	30	M	N/A	N/A	20/100	20/50	−0.50 +2.00 × 180°	Plano + 1.50 × 180°	N/A	N/A	70° (V4e) 65° (III4e)	70° (V4e) 65° (III4e)
B	MOGL 3138	67	45	M	N/A	N/A	20/150	12/400	−1.50 +1 × 180°	−1.50 +1 × 180°	N/A	N/A	70° with pericentral defect (V4e), peripheral island (I4e)	70° with patchy defects (V4e), 5° with peripheral island (I4e)
C	13573-BD	47	30	F	20/100 (age 38)	20/200 (age 38)	1/30	LP	−4.75 −1.75 × 95°	−5.0 −1.5 × 90°	Concentric narrowing ;to 5° with target III4e, peripheral residual slim islands (age 38)		residual islands peripheral (target V4e), central island <3°	
D	3338-BD	54	24	F	N/A	N/A	HM	HM	−1.75 −0.5 × 76°	−0.75 −1.0 × 66°	N/A	N/A	Residual islands peripheral (target V4e)	
E	3549-BD	41	20	F	20/20	20/20	20/20	20/20	−1.25 −0.5 × 105°	−1.0 −0.75 × 77°	N/A	N/A	Normal outer boundaries with III4e, decreased sensitivity and “patchy” defects with I4e and I3e targets	
F	KW	49	31	F	20/40	20/40	HM	20/400	Plano	Plano	Normal peripheral limits, pericentral sensitivity loss (I2 not seen), central relative scotoma (I3 and I4), enlarged blind spot	Normal peripheral limits, pericentral sensitivity loss (I2 not seen), enlarged blind spot	Mild concentric constriction, central scotoma (V4), residual temporal crescent (III4), I2 and I3 not seen	Mild concentric constriction, central scotoma with small preserved patch nasal to blind spot (V4)
G	10906-BD	77	57	F	20/50 (age 75)	20/200 (age 75)	20/100	20/400	+1.0 −2.0 × 110°	±0	N/A	N/A	N/A	N/A
H	RCD	38	30	F	20/30	20/25	20/40	20/30	Plano	Plano	N/A	N/A	Mild concentric constriction, enlarged blind spot (V4) considerable sensitivity loss, doughnut shaped annular scotoma (I4), I2 and I3 not seen	Considerable concentric constriction, enlarged blind spot (V4) considerable sensitivity loss, doughnut shaped annular scotoma (I4), I2 and I3 not seen
I	11431-USHII	33	21	F	20/25 (age 22)	20/40 (age 22)	20/40	20/50	−3.25 −1.25 × 7°	−4.0 −1.5 × 174°	Concentric narrowing to 40° with target III4e (age 22)		Concentric narrowing to 10°, no peripheral islands (target III4e)	
J	6284-BD	51	18	M	20/20	20/20	20/40	20/40	Plano	Plano	Concentric reduction, ring scotoma and residual central visual field island of central 5°	Constriction of peripheral visual field, ring scotoma, finally residual central island of central 5°	N/A	N/A

	Symptoms			Morphology			
Family	Nyctalopia	Photophobia	Color vision defect	Cornea	Lens	Macula	Peripheral Retina
A	Y	N	N	Clear	Early cortical changes OS	Relative foveal sparing	Areas of RPE atrophy and choroidal sclerosis, normal vessel caliber, peripheral bony spicules
B	Y	N	N	Clear	PSCC	Macular involvement	Crystals in periphery and posterior pole, choroidal sclerosis, tigroid
C	Y	Y	N	Clear	Cortical Opacities	Atrophic	Optic disks vital, narrow vessels, generalized RPE atrophy, crystalline deposits
D	Y	N	N	Limbus: crystalline deposits	Crystalline deposits, PSCC	Huge macular hole with neurosensoric detachment on OD, macula atrophic OS	Optic disks vital, narrow vessels, generalized RPE atrophy, crystalline deposits
E	Y	N	Y	Clear	Clear	None commented	Optic disks vital, peripapillary atrophy, vessels moderately attenuated, posterior pole with patchy RPE-atrophy and crystalline deposits, some pigment clumps
F	Y	N	Y (severe R/G; severe B/Y)	Very small peripheral crystals	Mild lenticular sclerosis	Extreme outer retinal and choriocapillaris atrophy, small white inner retinal crystals	Patchy outer retinal and choriocapillaris atrophy with fine white retinal crystals mostly in midperiphery, spicular inner retinal pigment migration with some larger pigment patches, small scalloped patches of preserved retina in periphery
G	Y	Y (mild)	N/A	Clear	Pseudophakic	Severe atrophy, crystals and yellow white flecks	Pigmentary changes
H	Y	N	Y (medium R/G defect; severe B/Y)	Clear	Clear	Patchy outer retinal and choriocapillaris atrophy with fine white inner retinal crystals	Patchy outer retinal and choriocapillaris atrophy with fine white retinal crystals mostly in midperiphery, spicular inner retinal pigment migration, with some larger pigment patches
I	Y	Y	Y	Clear	Cortical opacities	Macular edema and inferior gliosis	Optic disks vital, narrow vessels, RPE atrophy, crystalline deposits
J	Y	Y	Y	Crystalline deposits	Initially clear, finally primarily subcapsular post cataract	Diffuse crystalline deposits and reduced reflexes	Crystalline deposits, progressive atrophy during follow up, finally choroideremia-like fundus

Family	Electrophysiology	OCT/FAF	Other ocular	Other systemic
A	Cone: 25% residual function, rod: 20% residual function	FAF: Patchy hypofluorescence OCT: intraretinal and subretinal crystals and edema	N/A	N/A
B	Cone: 5uV out of 120uV b wave, Rod: 12uV out of 220uV b wave	Foveal thinning, remodeling, intraretinal and subretinal crystals	N/A	N/A
C	Non recordable	Retinal atrophy, crystalline deposits	N/A	N/A
D	Non recordable	OD: huge macular hole and neurosensoric detachment OS: retinal atrophy, crystalline deposits	N/A	Depression, elevated blood pressure, elevated cholesterol levels
E	Ganzfeld: scotopic and photopic responses on the lower normal limits, somewhat delayed IT; mfERG: central responses with almost normal amplitudes and IT, in outer rings IT delayed, amplitude subnormal	Retinal atrophy, crystalline deposits, central retina (fovea) with almost intact photoreceptors	Patient notes central vision defects and difficultyreading	N/A
F	ERG: absent rod-specific and cone-specific responses	Outer retinal atrophy with fine retinal crystals at all levels, crystals most visible on infrared and redfree reflectance imaging, blue light fundus autofluorescence virtually absent	N/A	N/A
G	Full-field ERG: Dark adapted ERG 55% of normal, light adapted 45% of normal, flicker 40% of normal; mfERG: severe central amplitude reduction	OCT not available, FAF multiple patchy areas of intensity loss	N/A	N/A
H	ERG: absent rod-specific responses; residual cone-specific responses	Outer retinal atrophy with fine retinal crystals at all levels, crystals most visible on infrared and redfree reflectance imaging, blue light fundus autofluorescence shows hypofluorescent scalloped patches surrounded by thin interconnecting bridges of normo- and hyperautofluorescence	N/A	N/A
I	Ganzfeld ERG extinguished, mfERG central emaining responses	Cystoid macular edema, crystalline deposits	N/A	Hearing problems, thalassemia minor, chronic nephropathy
J	Initially negative scotopic ERG. Finally nondetectable.	Deposits in deep retinal layer, obscured in fluorescein angiography	N/A	N/A

**Figure 4:**
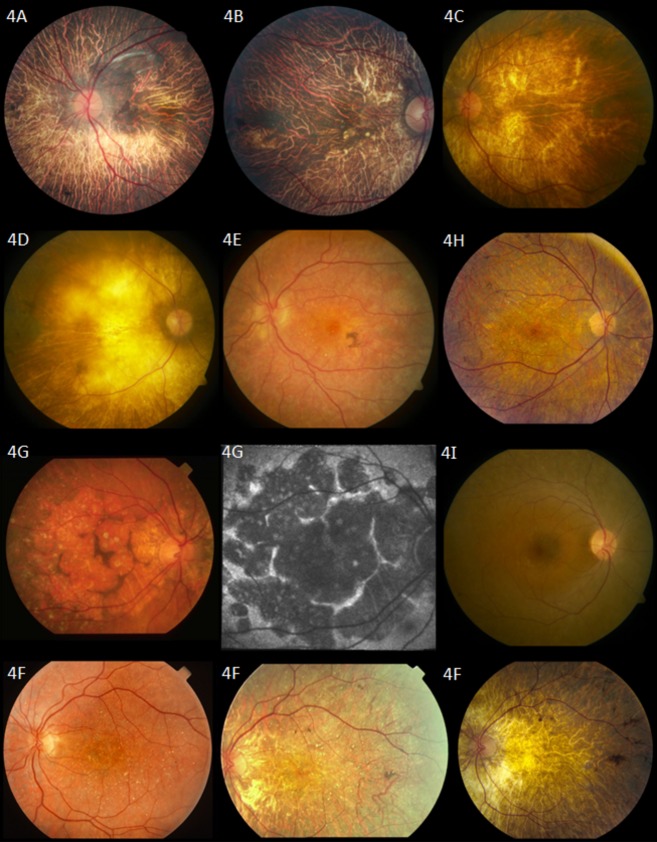
Fundus photographs. Proband A: 57-year-old patient with homozygous mutations in *CYP4V2* p.(Ile111Thr). Fundus photograph shows choroidal sclerosis and peripheral pigmentation. No crystal deposits are seen in this patient's fundus. Proband B: 67-year-old patient with homozygous mutations in *CYP4V2* p.(Glu202Lys). Fundus photograph shows severe choroidal sclerosis involving the entire macula and fovea, narrowing of the retinal vasculature, with relatively normal optic nerve appearance. Several crystalline deposits can be seen in the macula. Proband C: 47-year-old patient with homozygous mutation in *CYP4V2* c.604+4A>G; p.(?). Severe choroidal sclerosis and crystalline deposits in macula. Proband D: 54-year-old patient with compound heterozygous mutations in *CYP4V2* c.802-8_810delinsGC; p.(?), p.(Arg400Cys). Severe choroidal sclerosis and area of visible sclera. Few crystalline deposits seen in midperiphery. Proband E: A 41-year-old patient with compound heterozygous mutations in *CYP4V2* p.(Thr81Arg), p.(Arg400Cys). Mild choroidal sclerosis, crystalline deposits, and pigment clump. Some maculopathy is seen. Retinal vasculature remains relatively normal. Proband F: 49-year-old patient with homozygous mutations in *CYP4V2* p.(Thr417Nfs*2). Fundus photographs show progression of disease in left eye (left to right). Of note is the progressive choroidal sclerosis, vascular attenuation and decreasing presence of crystalline deposits over time. Photos taken at ages 31, 38, and 49, respectively. Proband G: 77-year-old patient with compound heterozygous mutation in *CYP4V2* c.802-8_810delinsGC; p.(?), p.(Arg400Cys). Unusual fundus appearance (left) showing large, similarly-sized discoid patches of atrophy separated by small ridges of intact tissue with presence of crystals and choroidal sclerosis. Optic disk and retinal vasculature appear relatively normal. Fundus autofluorescence (middle) confirms areas of atrophy with patches of decreased autofluorescence surrounded by walls of lipofuscin metabolism. Proband H: 38-year-old patient with homozygous mutations in *CYP4V2* p.(Arg465Gly). Choroidal sclerosis with diffuse crystalline deposits and pigment clumps in midperiphery. Proband I: 33-year-old patient with one heterozygous mutation in *CYP4V2* p.(Ile111Thr). Fundus photograph reveals nondescript retina with few crystalline deposits.

**Figure 5:**
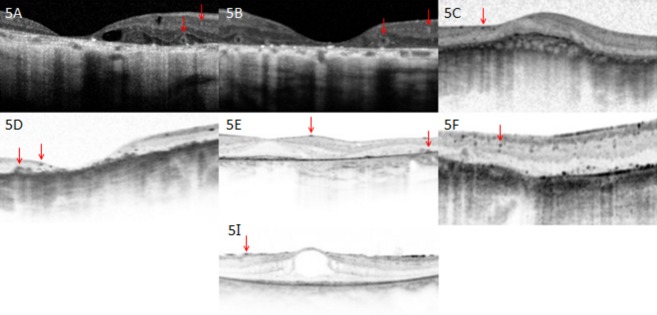
Optical coherence tomography images. Proband A: 57-year-old patient with homozygous mutations in *CYP4V2* p.(Ile111Thr). Optical coherence tomography shows severe foveal thinning and cystoid macular edema. Of note are two types of crystal that can be seen; tiny intraretinal crystals and large encapsulated subretinal crystals (red arrows). Proband B: 67-year-old patient with homozygous mutations in *CYP4V2* p.(Glu202Lys). Shown are remarkable thinning of the fovea and remodeling of retina, as well as presence of both intraretinal and subretinal crystals. Proband C: 47-year-old patient with homozygous mutation in *CYP4V2* c.604+4A>G; p.(?). Remodeling of retina with almost complete loss of architecture is seen, as well as tiny intraretinal crystals. Proband D: 54-year-old patient with compound heterozygous mutations in *CYP4V2* c.802-8_810delinsGC; p.(?), p.(Arg400Cys). Optical coherence tomography shows severe foveal thinning, remodeling of the retina, as well as both intraretinal crystals and larger subretinal crystals. Proband E: A 41-year-old patient with compound heterozygous mutations in *CYP4V2* p.(Thr81Arg), p.(Arg400Cys). Presence of both intraretinal and subretinal crystals. Photoreceptors in fovea remain intact. Proband F: 49-year-old patient with homozygous mutations in *CYP4V2* p.(Thr417Nfs*2). Optical coherence tomography shows fine intraretinal crystals. Proband I: 33-year-old patient with one heterozygous mutation in *CYP4V2* p.(Ile111Thr). Optical coherence tomography reveals presence of intraretinal crystals.

The predominant findings on fundus examination were extensive choroidal sclerosis, crystalline intraretinal deposits, attenuated vessels, and intraretinal spicular pigmentation in varying degrees of severity (Fig.4). Macular atrophy was often seen. Of note is proband A, whose fundus examination revealed choroidal sclerosis and peripheral pigmentation but no crystal deposits. On OCT, however, crystal deposits were observed in the subretinal space (Figs.4A and 5A). Proband J was similarly reported to have a “choroideremia-like” fundus appearance, with deposits seen in the subretinal layer on OCT. Proband G was also noted to have an unusual fundus appearance, consisting of large discoid patches of atrophy separated by ridges of relatively intact tissue, along with the presence of choroidal sclerosis and crystalline deposits (Fig.4G).

On OCT, two types of crystals were visualized in patients – tiny intra- and inner retinal crystals and larger, encapsulated subretinal crystals. All nine patients who had OCT were noted to have fine inner retinal crystals; five among these patients also showed larger subretinal crystals (Fig.5A, B, D, and E, no image available for proband J). Foveal thinning and remodeling of retinal architecture were other pertinent findings. In addition, cystoid macular edema (CME) was documented in two patients. ERG was abnormal in all patients who underwent testing, ranging from nonrecordable to subnormal photoreceptor function.

Disease progression was noted in most patients for whom previous clinical data were available, with declining VA and visual fields. Progression of disease in the fundus of proband F over 18 years was documented photographically (Fig.4F) and shows the development of the choroidal sclerosis and the clinical disappearance of the retinal crystals.

The clinical presentation of patients without identified *CYP4V2* mutations was indistinguishable from that of the *CYP4V2*-positive patients. The phenotypes of five of the *CYP4V2*-negative patients are shown in Figures S1 and S2. These patients demonstrated the same severe changes that were present in the patients with *CYP4V2* mutations, including choroidal show and sclerosis, the diffuse nature of disease, bone spicules, and macular involvement with or without crystalline deposits. OCT images similarly reveal the fine inner retinal crystals as well as the larger sometimes encapsulated subretinal crystals.

## Discussion

We identified eight different *CYP4V2* mutations in 10/19 patients in our cohort of crystalline retinal dystrophies, clinically diagnosed as Bietti crystalline dystrophy. We identified four novel mutations and one genomic rearrangement (deletion). This is the first large deletion reported for BCD. Eight patients were found to have two mutations in *CYP4V2*, while in two patients only one mutation was detected, despite extensive analysis of the coding region using Sanger sequencing and CNV analysis. Until now, 57 mutations in *CYP4V2* have been associated with BCD. These include 43 missense/nonsense mutations, eight splicing mutations, four small deletions, one small insertion, and one small indel (Li et al. 2004; Lee et al. 2005; Lin et al. 2005; Shan et al. 2005; Wada et al. 2005; Jin et al. 2006; Lai et al. 2007; Zenteno et al. 2008; Mamatha et al. 2011; Xiao et al. 2011; Yokoi et al. 2011; Haddad et al. 2012; Manzouri et al. 2012; Parravano et al. 2012; Song et al. 2013; Halford et al. 2014; Yin et al. 2014). To our knowledge, we are the first to identify a deletion including *CYP4V2* and several other genes in a patient with BCD, expanding the molecular pathogenesis of BCD. Interestingly, previous studies have reported five patients in which only single heterozygous mutations were found in *CYP4V2*, possibly implicating *CYP4V2* CNVs as found in proband F (Li et al. 2004; Shan et al. 2005; Jin et al. 2006; Rossi et al. 2013).

The pathogenic role of CNVs and the importance of their detection has been previously described in recessive retinal dystrophies such as retinitis pigmentosa, Leber congenital amaurosis (LCA), and cone dystrophy with supernormal rod response (CDSRR) (Wissinger et al. 2011; Eisenberger et al. 2013). Ultimately, the detection of such CNVs clarifies diagnosis, and may influence genetic counseling. Our results demonstrate that CNV is a mutational mechanism that can lead to BCD as well, and that screening for such variations may be necessary to avoid diagnostic uncertainties.

The deletion in proband F also covers two genes *KLKB1* and *F11*, both of which are involved in the contact activated coagulation pathway. *KLKB1* encodes plasma prekallikrein – also known as Fletcher factor – a glycoprotein in the kallikrein–kininogen–kinin system (KKS) which participates in coagulation, fibrinolysis, and inflammation. Plasma prekallikrein is converted to the serine protease plasma kallikrein by factor XIIa through cleavage of an internal Arg-Ile bond (Chung et al. 1986). Kallikrein then cleaves both low- and high-molecular-weight kininogens to release bradykinin and lys-bradykinin, mediating the effects of the KKS (Sainz et al. 2007). Recessively inherited mutations in *KLKB1* are known to cause plasma prekallikrein deficiency, resulting in a prolonged activated partial thromboplastin time (aPTT) without increased bleeding tendency in affected patients (Lombardi et al. 2003). Heterozygous patients have been reported to have decreased prekallikrein activity but normal aPTT values (Wynne Jones et al. 2004).

Factor XI is a homodimeric glycoprotein that circulates in plasma as a noncovalent complex with high-molecular-weight kininogen (Fujikawa et al. 1986). Factor XI is activated by factor XII, *α*-thrombin, and factor XI (autoactivation); it participates in contact activated coagulation by catalyzing the conversion of factor IX to factor IXa and thereby sustaining thrombin generation (Naito and Fujikawa 1991). Factor XI deficiency (sometimes referred to as “hemophilia C”) is a rare mild-to-moderate bleeding disorder associated with mutations in *F11*. Both autosomal recessive and dominant modes of inheritance have been described, the latter possibly being secondary to dominant-negative mutations resulting in mutant proteins forming nonsecretable heterodimers with wild-type subunits (Kravtsov et al. 2004). Moreover, earlier studies demonstrated considerable variability in rates of bleeding in heterozygotes, with some studies even noting no distinction between homozygotes and heterozygotes (Ragni et al. 1985; Bolton-Maggs et al. 1988). Upon revisiting proband F's medical records, we found no history of coagulopathy.

In addition to the two patients in whom only single mutations were detected, we failed to identify any *CYP4V2* mutations in nine other patients. The rate of *CYP4V2* mutation detection demonstrated in our study was relatively low, especially when compared to a previous study by Xiao et al. (2011), in which *CYP4V2* mutations were found in >95% of BCD patient families (Xiao et al. 2011). Our results suggest that *CYP4V2* mutations in our patients may reside in genomic locations that were not studied in our gene sequencing protocol, that is, in promoter, untranslated region, or deep intronic regions. Alternatively our data suggests locus heterogeneity and a second Bietti's gene. We were able to re-examine five *CYP4V2*-negative crystalline retinal dystrophy patients and found that their phenotypes (Figs. S1, S2) are indistinguishable from our BCD patients.

In accordance with previous studies, we found considerable phenotypic variability in our cohort, with no obvious correlation of severity or features with patient demographics. Although disease progression was noted in most patients (for whom historical clinical data were available), length of progression was not clearly associated with a more severe phenotype. Previously, it has been postulated that environmental factors affecting lipid metabolism (e.g., diet) may also contribute to the spectrum of disease (Lee et al. 2005; Rossi et al. 2013). Significant clinical variability was also noted for patients sharing common genotypes, and even within one family. However, it can be observed that in general within our cohort, those with genotypes resulting in splicing defects or nonsense mutations (i.e., c.802-8_810delinsGC; p.(?), c.604+4A>G; p.(?), and c.1249dup; p.(Thr417Asnfs*2)) demonstrated greater clinical severity. Notably, proband F with the genomic deletion was documented to have one of the most clinically severe phenotypes in our cohort. It is possible that the heterozygous *CYP4V2* deletion contributes to her advanced disease state, however this remains unclear as the patient was also found to have a severe frameshift mutation.

Furthermore, proband E, with a mild phenotype despite 21 years of disease, was found to have compound heterozygous mutations, with one allele c.242C>G; (p.Thr81Arg) predicted to be comparatively benign (Table2).

It should be noted, however, that these correlations between mutation and disease severity have not been consistently shown in previous studies; for instance, although Lai et al. (2007) and Halford et al. (2014) document a similar pattern, Rossi et al. (2013) note that this correlation was not present in their patients. As well, two patients (one with mutation c.1526C>T; (p.P509L) and the other c.1393A>G; (p. p.R465G)) in whom only single mutations were identified in previous studies failed to show the same severity of disease as seen in proband F (Jin et al. 2006; Rossi et al. 2013). The clinical features of the three other heterozygous patients in the literature were not described (Li et al. 2004; Shan et al. 2005).

In our patients, we documented four locations and types of the crystals: small, widespread inner retinal crystals; large, encapsulated subretinal crystals; corneal limbus crystals; and lenticular crystals. The presence of encapsulated subretinal crystals (in addition to inner retinal crystals) on OCT has previously been reported (Pennesi and Weleber 2013). Initially described by Zweifel et al. (2009) in a series of patients with various retinal disorders, these structures were termed “outer retinal tubulation.” However, a more recent study with BCD patients found that the hyperrefractive structures identified were in fact spherical (Kojima et al. 2012).

It is of note that proband D with compound heterozygous mutations c.802-8_810delinsGC; p.(?) and c.1198C>T; p.(Arg400Cys) was found to have crystalline deposits on the lens, which is atypical for BCD. Two separate reports of lenticular crystals have been documented previously, with both patients described carrying the c.802-8_810delinsGC mutation as well, though in a homozygous state (Yokoi et al. 2010; Chung et al. 2013).

The phenotype of BCD is progressive and severe. In the later stages, as retinal crystals become increasingly difficult to detect on clinical examination and disappear with disease progression, the phenotype can overlap with choroidal sclerosis and some phases of choroideremia; for instance, as reported in the late-stage fundus appearance of probands A and J (Mataftsi et al. 2004; Mansour et al. 2007; Xiao et al. 2011). It is therefore likely that when patients are seen in the later stages of disease, a diagnosis of a nonspecific retinal dystrophy or atypical retinitis pigmentosa may be made. However, in both these patients we documented that OCT reveals nevertheless the presence of retinal crystals. Our results therefore indicate that OCT plays an essential role in the diagnosis and differentiation of BCD from other retinal pathology presenting primarily with choroidal sclerosis.

In conclusion, we identified four novel *CYP4V2* mutations in 10/19 BCD patients as well as the first genomic rearrangement (large deletion) implicating *CYP4V2*. Our results emphasize the importance of CNV screening in BCD. Although the phenotype of the patient with the *CYP4V2* deletion was severe, no clear genotype–phenotype correlation could be established. It would be interesting to note what patterns emerge in future when more patients with large deletions as such are reported. Finally, the nine patients in whom no *CYP4V2* mutations were found suggest that *CYP4V2* mutations may reside in genomic locations that were not covered by our protocol. Alternatively, locus heterogeneity might underlie BCD; further investigations will follow to explore these possibilities.

## Supporting Information

Figure S1 Fundus photographs of *CYP4V2*-negative patients. Shown are fundus photographs of five *CYP4V2*-negative Bietti crystalline dystrophy patients. As in those with identified *CYP4V2* mutations, predominant findings include choroidal sclerosis with or without visible crystalline deposits, pigment deposition, and vascular attenuation.

Figure S2 Optical coherence tomography images of *CYP4V2*-negative patients. OCT images of five *CYP4V2*-negative patients, with presence of small hyperreflective intraretinal crystals and larger subretinal crystals (red arrows).

Table S1 *CYP4V2* primer sequences.

Table S2 Conditions and primer sequences.
