# Supplementary figures and images for: Novel insights into the molecular pathogenesis of CYP4V2-associated Bietti's retinal dystrophy
Source: Mol Genet Genomic Med. 2014 Sep 15;3(1):14–29. doi: 10.1002/mgg3.109 (PMC4299712; doi:10.1002/mgg3.109)

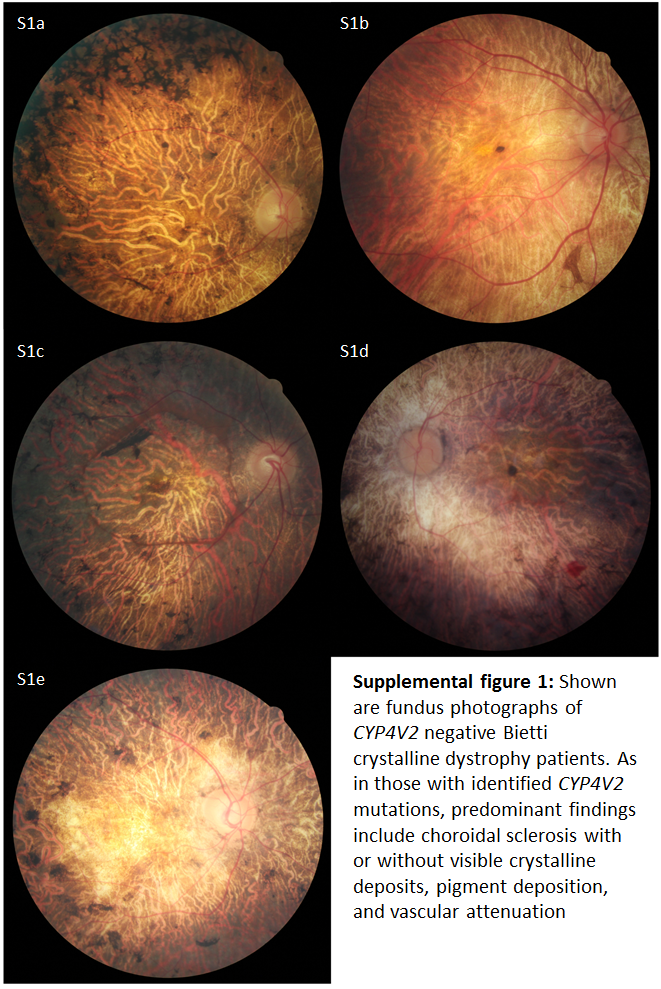

Supplement: Figure S1 — Fundus photographs of CYP4V2-negative patients. Shown are fundus photographs of five CYP4V2-negative Bietti crystalline dystrophy patients. As in those with identified CYP4V2 mutations, predominant findings include choroidal sclerosis with or without visible crystalline deposits, pigment deposition, and vascular attenuation. [file mgg30003-0014-sd1.tif]

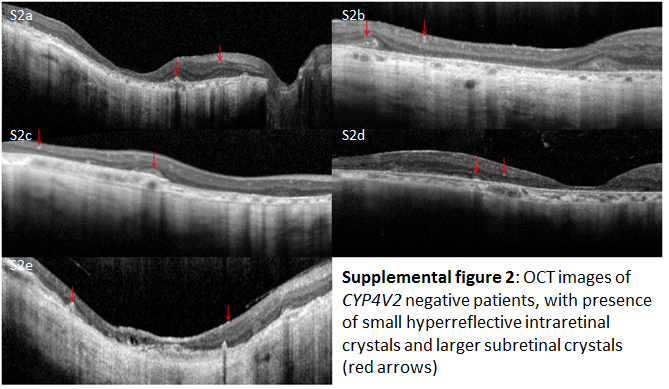

Supplement: Figure S2 — Optical coherence tomography images of CYP4V2-negative patients. OCT images of five CYP4V2-negative patients, with presence of small hyperreflective intraretinal crystals and larger subretinal crystals (red arrows). [file mgg30003-0014-sd2.tif]
