# Supplementary material for: Novel insights into the molecular pathogenesis of CYP4V2-associated Bietti's retinal dystrophy
Source: Mol Genet Genomic Med. 2014 Sep 15;3(1):14–29. doi: 10.1002/mgg3.109 (PMC4299712; doi:10.1002/mgg3.109)
Supplement: Table S1 — CYP4V2 primer sequences. [file mgg30003-0014-sd3.doc]

**Supplemental table 1: *CYP4V2* primers**

| **Exon** | **Primer forward (5’-3’)** | **Primer reverse (5’-3’)** | **Product size (bp)** |
| --- | --- | --- | --- |
| 1 | GAAACGTCGTTCCGGGG | TGACAGGCTCCTCTCTCCTG | 482 |
| 2 | GAGAAAACAAACCTTTGTCCAATAC | AGGACACGTTATATTGATTCTGG | 273 |
| 3 | GGAGAAAATAAATGTTGTGAATGC | TTCTTGAAATAACAAGTTGCACG | 278 |
| 4 | TGCTTTAATCGTTTTGGATGTTAC | GTGTGATTTCCTGTTTGGGC | 345 |
| 5 | CGCTGCAAAATAAACACGAG | GATACAACGCAGAAATTGTTAGC | 224 |
| 6 | GACAATCATCGTCATTCCCAC | TGCACTTAATACCACCAAACTG | 336 |
| 7 | TCACAAGAGCCTATGTTGTCG | AAGAAGTTGAGCTGGTACTTAATCAG | 506 |
| 8 | TCACTCCTAATCATCGCAGC | GCCTTCCTGCTCATTACACTG | 256 |
| 9-10 | ATGCCTTGATCCACCTGTTC | CACTGTGAGAAACCCACCATC | 559 |
| 11 | TCTTGTTTCTCACATTTGGGG | TTAGGTCTAGGGGATTCAAGC | 348 |
