# Supplementary material for: Novel insights into the molecular pathogenesis of CYP4V2-associated Bietti's retinal dystrophy
Source: Mol Genet Genomic Med. 2014 Sep 15;3(1):14–29. doi: 10.1002/mgg3.109 (PMC4299712; doi:10.1002/mgg3.109)
Supplement: Table S2 — Conditions and primer sequences. [file mgg30003-0014-sd4.docx]

**Supplemental table 2: Conditions and primer sequences**

| **Protocol SsoAdvanced (for gDNA)** | | |
| --- | --- | --- |
| Enzyme activation | 98°C-->2min |  |
| Amplification | 98°C-->5sec | 40x |
|  | 60°C-->30sec |  |
| Smeltpiek |  |  |
| Cooling |  |  |

| **Primers** | **Forward** | **Reverse** |
| --- | --- | --- |
| CYP4V2_CNV_1 | CGCCAGTCTGGTCCTGAG | TGATCGGAGGGTGGGAAC |
| CYP4V2_CNV_2 | TGCTCTCTACCTGGCTTCCT | TTCTGGGGAAAAACATCAAA |
| CYP4V2_CNV_3 | TGTATGTCTCTAAAGTATGTTTTTCTCTTC | GACAGCTTTACAAAGGTACACTGG |
| CYP4V2_CNV_4 | GCTCCAGGAGAAAGATGTTA | ATCTAAGGCACAAAGAGTGAT |
| CYP4V2_CNV_5 | GTCTGCCGCTGCAAAATAA | TTTACCTATAAACTGCACGGACA |
| CYP4V2_CNV_6 | GAGTGAGATGATATTTCGAAGAATAAAG | ACATAAGGTACCAGAGATCAAG |
| CYP4V2_CNV_7 | AGTCCAAACAGAAGCATGTGA | AAGGCCCTGCGTTTATTTTT |
| CYP4V2_CNV_8 | TATTGTTTTCTGCATTTGTAGGG | GGGTTAGAACCCAACAGGTATAA |
| CYP4V2_CNV_9 | CCCACTGCTCTTTCAGGTC | TCCAGATACCGAAGTTTCTTCA |
| CYP4V2_CNV_10 | AGGAGTTCCAGCCTGAGC | CATCAAGTGGGCCTGAAAG |
| CYP4V2_CNV_11 | TGGTCTAGAAGGACAGTTGA | AGATCAGTGGGATCAAGGAA |

| **Mix qPCR** | **1 reaction** |
| --- | --- |
| 2x mastermix Bio-Rad | 2,5 µl |
| Forward primer (5 µM) | 0,25 µl |
| Reverse primer (5 µM) | 0,25 µl |
| DNA template (10 ng/µl) | 2 µl |
